# Dual RNA isolation from blood: an optimized protocol for host and bacterial RNA purification for dual RNA-sequencing analysis in whole blood sepsis samples

**DOI:** 10.1099/mgen.0.001501

**Published:** 2025-09-18

**Authors:** Isabella Anna Joubert, Christopher Mullally, Edward Litton, Edward Raby, Abha Chopra, Tobias Strunk, Penghao Wang, Andrew Currie

**Affiliations:** 1 School of Medical, Molecular and Forensic Sciences, Murdoch University, Perth, Australia; 2 Personalised Medicine Centre, Murdoch University, Perth, Australia; 3 Wesfarmers Centre of Vaccines and Infectious Diseases, The Kids Research Institute Australia, Perth, Australia; 4 Clinical Perinatal Research Laboratories, King Edward Memorial Hospital, Perth, Australia; 5 School of Medicine, University of Western Australia, Perth, Australia; 6 Intensive Care Unit, Fiona Stanley Hospital, Perth, Australia; 7 State Adult Burns Unit, Fiona Stanley Hospital, Murdoch, Australia; 8 Microbiology Department, PathWest Laboratory Medicine, Murdoch, Australia; 9 IIID, Murdoch Medical Genomics Core Laboratory, Precision Medicine Center, HFI, Murdoch University, Perth, Australia; 10 Neonatology, King Edward Memorial Hospital, Child and Adolescent Health Service, Perth, Australia; 11 Centre for Crop and Food Innovation, Food Futures Institute, Murdoch University, Perth, Australia

**Keywords:** bacterial virulence, dual RNA-sequencing, host–pathogen interactions, sepsis, transcriptomics

## Abstract

Dual RNA-sequencing (dual RNA-seq) holds significant promise for deciphering bacterial virulence mechanisms during systemic infections. However, its application in sepsis research is hindered by technical challenges, including a low bacterial burden in blood and limited sample volumes and RNA yield from vulnerable populations, such as neonates. We developed an optimized protocol [dual RNA isolation from blood (DRIB)] for simultaneous stabilization, isolation and purification of high-quality host leukocyte and bacterial RNA from low-volume whole blood samples (0.5 ml). This protocol is compatible with clinical sample collection workflows and high-throughput RNA sequencing. The feasibility of DRIB for dual RNA-seq was validated using a pilot cohort of clinical adult sepsis samples, enabling the investigation of host–bacterial gene expression during sepsis. The DRIB protocol yielded 2.10–6.91 µg of total RNA per clinical sample in our pilot cohort. Dual-species ribosomal RNA (rRNA) depletion and RNA-seq generated 16.6–24.8 million filtered reads per sample, with 63±7% of reads uniquely mapped to host or bacterial sequences. Host genes accounted for 51–68% (8.4–10.9 million) reads, while 0.5–6.7% (79,496–789,808 reads) mapped to bacterial genomes. Bioinformatic analysis revealed that both shared and individual transcriptional patterns were identified in host and bacterial responses, including pathways related to immune metabolism and metal-ion binding. Our optimized DRIB protocol and RNA-seq pipeline effectively captured both host and bacterial RNA transcription in clinical sepsis samples. Expanding this approach to larger cohorts and varying disease timepoints will provide crucial new insights into host–bacterial gene co-expression dynamics in sepsis progression and outcomes.

**Impact Statement**

Bacterial sepsis continues to pose a significant global health challenge, and despite advances in critical care, our understanding of the complex interplay between host immune responses and bacterial pathogens during sepsis remains limited. Our study introduces a novel approach leveraging dual RNA-sequencing to simultaneously investigate host and bacterial gene expression in blood samples of sepsis patients, representing a major advancement over traditional methods that typically focus on either host or pathogen alone. Using our optimized protocol, we successfully isolated high-quality RNA from low-volume (0.5 ml) clinical sepsis samples, capturing transcriptional data from both patients and their diverse bacterial pathogens. This methodology establishes a foundation for future research into the intricate dynamics of host–pathogen interactions, paving the way for improved insights into sepsis pathogenesis and potential therapeutic strategies for diverse patient populations.

## Data Summary

All sequence data generated by this study are available on public data repositories of the European Molecular Biology Laboratory European Bioinformatics Institute (EMBL-EBI, ID: E-MTAB-14713), which can be accessed at https://www.ebi.ac.uk/biostudies/arrayexpress/studies/E-MTAB-14713.

## Introduction

Bacterial sepsis describes a life-threatening clinical syndrome caused by a dysregulated host response to infection [1]. Due to the heterogeneous nature of sepsis, with varying causative pathogens and infectious origins, host- and pathogen-specific drivers of sepsis remain incompletely understood [2,4]. By simultaneously capturing host and pathogen transcriptomes, dual RNA-sequencing (dual RNA-seq) [5] can help dissect the molecular host–pathogen interplay during infection and identify key gene regulatory networks and potential therapeutic targets of invasive bacterial diseases [6,8].

However, the application of dual RNA-seq to sepsis research faces several challenges. Preterm infants, who are at high risk of developing sepsis [9], have limited accessible blood volumes (≤1 ml) for analysis [10,11]. The low bacterial burden typically observed in many blood samples from neonatal, paediatric and adult patients with sepsis [12,14] also poses a significant obstacle to comprehensive transcriptional analysis of the causative pathogen during infection [15]. These challenges are compounded by the need for a protocol that effectively lyses both host and bacterial cells (including Gram-positive organisms), preserving RNA from both species without degradation, while maximizing yield and purity from low-volume samples.

We addressed these challenges by optimizing a protocol for the effective recovery of high-quality host and bacterial RNA transcripts from low-volume whole blood samples (0.5 ml). The protocol is compatible with large-scale clinical sample collection and high-throughput RNA-seq. We optimized the protocol to ensure the recovery of Gram-positive bacterial RNA transcripts (*Staphylococcus epidermidis* and *Staphylococcus aureus*), given the clinical relevance of these species in the context of neonatal and biomedical device-related sepsis [9,16,18], as well as the added difficulty associated with lysing bacteria with resilient cell walls. We demonstrate our protocol’s capabilities for use with small-volume clinical sepsis samples (0.5 ml), explore transcription patterns in both host and pathogen in these samples, and lastly, discuss its potential in future applications to advance our understanding of sepsis pathogenesis.

## Methods

### Bacterial strain selection and growth conditions

*S. epidermidis* strain 1457, isolated from a venous catheter infection [19], along with *S. epidermidis* strain 12228, *S. aureus* strain 29523 and *Escherichia coli* strain 29522, obtained from the American Type Culture Collection (ATCC, Virginia, USA), were used in this study. Bacterial strains were recovered from frozen (−80 °C) bead stocks (TSC Protect) onto horse blood agar (HBA) plates (Thermo Scientific, HBA Columbia) as needed and incubated overnight at 37 °C. Single colonies were incubated in 10 ml sterile BBL^™^ trypticase^™^ soy broth (TSB; BD) statically overnight at 37 °C to form a primary liquid culture. The following day, 20 ml of sterile TSB was inoculated with primary liquid culture to reach an OD of ~0.06, and bacterial cultures were grown at 37 °C and 160 r.p.m. (Incubator Shaker 8500, BioLine) until the mid-logarithmic (mid-log) growth phase was reached (mean OD ~0.25 for *S. epidermidis* and OD ~0.34 for *S. aureus*, as determined by bacterial growth curves performed in TSB). Mid-log bacterial cultures were either used immediately or transferred into 2 ml cryo-vials (CRYO.S^™^, Greiner) in 1.2 ml aliquots with the addition of 15% (v/v) sterile glycerol (Sigma) for *E. coli* or 20% (v/v) heat-inactivated FCS (Sigma) for *Staphylococcus* spp. as per in-house protocols for improved Gram-positive viability [20] and frozen at −80 °C until further use.

To assess bacterial viability, fresh or frozen bacterial mid-log stocks were serially diluted 10-fold (10^3^–10^6^) in sterile PBS (Gibco^®^). Three 20 µl samples from each dilution were spotted onto a single HBA plate and incubated overnight at 37 °C. HBA plates with triplicate spots containing ~10–80 single colonies were counted, and the mean c.f.u. ml^−1^ was determined.

### Bacterial RNA isolation

To establish an effective baseline for dual RNA-seq from blood samples, we first optimized bacterial RNA extraction using single-species cultures. This initial benchmarking phase allowed us to test a broad range of commercially available RNA extraction kits, as well as acid-guanidinium-phenol-based protocols, in combination with various chemical and mechanical lysis strategies. These evaluations focused on identifying methods to maximize total bacterial RNA yield (ng per 10^6^ c.f.u.) while preserving RNA integrity. All extractions were conducted using either freshly grown or frozen mid-log bacterial stocks at a concentration of ~10^7^ c.f.u. ml^−1^. Detailed descriptions of each tested extraction protocol are provided in File S1, available in the online Supplementary Material. Total bacterial RNA yield was quantified using the Qubit^™^ Fluorometer 2.0 (Life Technologies) with the RNA High Sensitivity kit (Life Technologies). Additionally, RNA integrity was assessed using the TapeStation (Agilent).

### Blood sample collection, inoculation, and RNA stabilization

PAXgene^™^ Blood RNA solution (PreAnalytix, Qiagen/Becton Dickson) is frequently used for the collection, storage and transport of clinical blood samples at the bedside to stabilize intracellular RNA. To determine whether blood sample storage in PAXgene^™^ RNA solution affects the bacterial RNA yield and bacterial gene expression, we prepared inoculated blood samples with a defined dose of bacteria and processed samples either following a 2 h incubation in PAXgene^™^ RNA solution at RT or stored samples at −80 °C for 7 days prior to RNA extraction.

Peripheral blood samples were collected from healthy adults (18–65 years) by venepuncture into Li-Heparin tubes (BD Vacutainer^®^, 10 ml) and processed within 60 min of collection. Freshly grown or frozen mid-log bacterial cultures of *S. epidermidis* 1457 and *S. aureus* 29523 (~10^7^ c.f.u.) were washed twice with PBS, and the resulting cell pellets were resuspended in 50 µl of nuclease-free water (NFW, Sigma). 0.45 ml of heparinized blood was then added to the bacterial suspensions and mixed carefully. Samples (0.5 ml) were immediately stabilized with 2.76× volumes (1.38 ml) of PAXgene^™^ Blood RNA solution in accordance with an adapted protocol previously established by our group for small-volume blood samples [21]. Samples (1.88 ml) were incubated at RT for 2 h to stabilize intracellular RNA and allow complete lysis of host erythrocytes and then processed or immediately frozen at −80 °C and incubated at RT for 2 h after thawing.

### Host–bacterial RNA isolation and purification (‘DRIB protocol’)

Based on the outcomes of our protocol optimization experiments, we developed a modified dual RNA isolation from blood (DRIB) protocol, suitable for low-volume clinical samples (Fig. S1) for the isolation of high-quality host and bacterial transcripts. Briefly, total RNA was extracted from 0.5 ml blood samples stabilized in PAXgene^™^ Blood RNA solution (1.88 ml total volume) as described previously. Samples were spun down (3,200 ***g***, 10 min), and the supernatant containing lysed host erythrocytes was carefully removed. Remaining cells (leukocytes and bacteria) were washed once with 1 ml NFW before cell pellets were dissolved in 100 µl NFW. 1 ml TRI reagent^™^ (Sigma) was added, and samples were transferred into 2 ml bead-beating tubes containing 0.1 mm zirconia/silica beads (PureLink^™^ Invitrogen^™^). Cells were lysed by performing 3×1 min cycles of bead-beating at 3,000 r.p.m. on a BioSpec Mini-Beadbeater-24 with 1 min breaks on ice in between lysing cycles. Samples were then incubated at RT for 5 min before phase separation was performed by adding 200 µl of chloroform (MP Biomedical), followed by a 2 min incubation at RT and centrifugation at 12,000 ***g*** for 15 min. The RNA-containing aqueous phase was collected and purified using the RNA Clean-and-Concentrator-25 kit (Zymo Research) according to the manufacturer’s instructions with a final elution volume of 30 µl to improve RNA recovery and concentrate the final sample for downstream analyses. 40 U of RNaseOUT^™^ Recombinant Ribonuclease Inhibitor (40 U µl^−1^, Invitrogen^™^) were added to eluted RNA samples before any remaining DNA traces were removed using the Ambion^®^ DNA-free^™^ DNase Treatment and Removal Reagents (Life Technologies) as per the manufacturer’s protocol.

### Targeted reverse transcription-quantitative PCR (RT-qPCR)

Forward/reverse primers and multiplex RT-qPCR probes for bacterial and human housekeeping genes, host defence genes, and bacterial virulence genes were designed in-house using the Geneious software (Dotmatics, v10.2.4) and synthesized by Integrated DNA Technologies (IDT, Coralville, IA, USA). The molecular characteristics of primers and probes are described in Table S5. A blastn search was conducted on sequences publically available on The National Center for Biotechnology Information (NCBI, https://www.ncbi.nlm.nih.gov/) database to ensure that the primer and probe sequences were specific to the transcripts of interest. Melting curve analysis and agarose gel visualization of PCR amplicons furthermore confirmed the specificity of designed primers for all target genes.

### cDNA synthesis and targeted RT-qPCR

Total RNA was reverse transcribed into cDNA using the QuantiTect Reverse Transcription kit (Qiagen) according to the manufacturer’s instructions using a consistent input volume of 12 µl purified RNA. cDNA samples were stored at −20 °C until RT-qPCR analysis. cDNA templates (2 µl) were amplified using the PerfeCTa qPCR ToughMix (Quantabio) with 300 nM of primers and 150 nM probe in a final reaction volume of 20 µl. The expression of *HUPO*, *B2M*, *IL1B* (*Homo sapiens*), *gyrB* and *psmβ1/2* (*S. epidermidis*) and *era* and *gyrB* (*S. aureus*) was measured in Multiplex RT-qPCR, and *secA* (*S. epidermidis*) and *hlgA* (*S. aureus*) were measured as Singleplex RT-qPCR reactions using a QuantStudio^™^ 6 Flex Real-Time PCR System (Applied Biosystems^®^). All assays were held at 95 °C for 5 min, followed by 40 cycles of 95 °C for 20 s and 55 °C for 30 s. All reactions were performed alongside no-template negative controls and positive controls (in-house human and bacterial genomic DNA templates).

### Clinical sepsis sample collection and RNA isolation

Hospitalized adult patients with a suspicion of sepsis referred for ICU admission at Fiona Stanley Hospital in Perth, Western Australia, were recruited to an ongoing prospective cohort sepsis study (The One Project Study, TOPS). Peripheral blood samples were collected from study participants by venepuncture into BACTEC^™^ Peds Plus^™^ (BD, 40 ml) and EDTA tubes (BD Vacutainer^®^, 10 ml). Within 2 h of blood sample collection, EDTA blood samples were aliquoted for transcriptional (0.5 ml), genomic (4 ml) and plasma metabolomic (5.5 ml) analyses. For this study, 0.5 ml EDTA blood samples were stabilized into pre-aliquoted PAXgene^™^ RNA solution (1.38 ml) and stored at −80 °C. Routine blood culture (BC) analysis was performed, and relevant data (BC result, culture conditions, causative pathogen, BC time-to-positivity, and sex) were obtained for this study. All blood samples were collected as close as possible to the routine BC sample. A subset (*n*=8) of BC-positive samples, collected between September 2021 and January 2023, was selected for dual RNA-seq based on the relevance of the causative pathogen and BC time-to-positivity. Host and bacterial RNA were purified from the TOPS clinical sepsis samples as described above using our optimized DRIB protocol. Total RNA was quantified, and RNA quality was assessed as previously described.

### rRNA depletion, cDNA library preparation, RNA-sequencing

Host and bacterial ribosomal RNA (rRNA) were depleted from purified total RNA samples using the Illumina Ribo-Zero Plus RNA Depletion kit (Illumina) following the manufacturer’s guidelines. The efficacy of dual-species rRNA depletion was verified using TapeStation. For cDNA library preparation for RNA-sequencing, an in-house adapted SmartSeq assay protocol [22] using oligoT and random hexamer primers was employed. Full-length cDNAs were amplified, purified and quantified using the Promega Quantus^™^ Fluorometer. The dscDNA was prepared for sequencing using the NEBNext^®^ Ultra^™^ II FS DNA Library Prep kit for Illumina (New England Biolabs). Sequencing was performed on a NovaSeq 6000 platform using 150 bp paired-end chemistry (Illumina Inc.).

### Bioinformatic analyses

#### Read processing and filtering

The raw sequencing reads were quality checked by FastQC v0.12.1 [23] and MultiQC v1.18 [24] following several criteria, including the sequencing quality score, G+C content distribution, and base call quality. Potential contamination and sequencing adapter content were screened by evaluating over-presented sequences using an in-house complete Illumina sequencing adapter database. FastQC/MultiQC analysis revealed that reads have been quality filtered by Illumina commercial software, and no further filtering was performed, as further processing by Trimmomatic v0.39 [25] could not improve results. Bioinformatic rRNA filtering was performed using RiboDetector v0.2.8 [26], a bi-directional long short-term memory neural network-based method, which was run in CPU mode with ensure parameter, which makes sure the classification has high confidence for paired-end reads.

#### Read alignment

Bacterial genomic sequencing reference and annotation files were created by collating known bacterial gene sequences obtained from NCBI databases using the ‘TaxaID’ of each of the seven causative bacterial species (*S. epidermidis*, *S. aureus*, *E. coli*, *Serratia marcescens*, *Streptococcus pneumoniae*, *Streptococcus dysgalactiae* and *Klebsiella pneumoniae*) detected in the clinical samples by routine BC. The human whole-genome reference and annotation file were obtained from the NCBI repository (version Hg38).

Filtered clean total RNA-seq reads were aligned to the bacterial references and human reference using RNA-STAR v2.7.1a [27] with the following options: (i) gene counting was selected with *quantMode GeneCounts*; (ii) up to 20 multiple site alignments are considered for alignment, with only unique mappings considered; (iii) alignment will be output only if the number of matched bases is higher than or equal to 0.3 normalized to the sum of paired-end reads’ lengths; (iv) alignment will be accepted only if the alignment score is higher than or equal to 0.3 normalized to the sum of paired-end reads’ lengths.

The alignment was performed in a two-step approach. The first alignment was performed using only bacterial transcriptome references, and the second alignment was completed using only the human genome reference. The gene counting/feature mapping was achieved by using RNA-STAR’s gene counting function and subread v2.0.6 [28] in pair-ended mode. Host and bacterial read count matrices were analysed separately in R (v4.3.1) [29].

#### Globin RNA removal

Globin gene RNA reads (*HBA1*, *HBA2*, *HBB*, *HBBP1*, *HBD*, *HBE1*, *HBG1*, *HBG2*, *HBM*, *HBQ1*, *HBZ*, *HBZP1*) were removed bioinformatically as previously described [30].

#### RNA-seq read filtering

Potential influential outlier samples were identified by quantifying dissimilarity between samples using L1 distance and Tukey’s method, utilizing the Kolmogorov–Smirnov test and the Hoeffding D statistic. A multiset Jaccard Index-based method was used to filter low-count genes [31].

#### Differential gene expression analysis

Unstimulated blood samples (0.1 ml) of eight healthy adults (18–25 years old) were collected and processed as previously described and included in the same sequencing run as clinical sepsis samples. Filtered gene expression data were normalized in R using the *DESeq2* (v1.40.2) [32] median-of-ratios approach. Differentially expressed host genes (DEGs) were determined by comparing clinical sepsis samples to each other (Gram-positive and Gram-negative causative pathogens), as well as to the unstimulated control samples. DEGs with Benjamini–Hochberg adjusted *P* values <0.05 and log2 fold changes (FC) of >2 were identified as significant. A Venn-Euler diagram was created using the *Eulerr* R package (v7.0.0) to show the overlap of pathogen-specific DEGs compared to the unstimulated controls.

#### Pathway enrichment analysis

Reactome pathway analysis was performed using the 100 most highly expressed host genes in each sepsis condition (Gram-positive/Gram-negative) using *Enrichr* [33,35], which uses Fisher’s exact test and the hypergeometric test to determine *P* values. *Q* values represent Benjamini–Hochberg adjusted *P* values.

#### Weighted gene co-expression network analysis

Weighted gene co-expression network analysis (WGCNA) was used to identify modules of co-expressed genes and their association with clinical traits using the *WGCNA* [36] and *impute* [37] R packages. The process involved normalizing the gene expression data using variance-stabilizing transformation (VST) from the *DESeq2* R package, followed by the construction of a signed network where the optimal soft-thresholding power was determined to achieve a scale-free topology. Modules were identified using the *blockwiseModules* function with a maximum block size of 10,000 genes. Linear models fitted to module eigengenes (MEs) were then correlated with clinical traits including ‘Status’ (sepsis vs. unstimulated controls), ‘Condition’ (Gram-positive, Gram-negative and unstimulated controls) and ‘Causative Pathogen’, using the *limma* (v3.56.2) R package [38] for empirical Bayes smoothing and statistical testing.

#### STRING protein association network analysis

Search Tool for the Retrieval of Interacting Genes (STRING) v12.0 (http://string-db.org) [39,40] was used to identify functional protein association networks for the 100 most highly expressed *E. coli* and *St. pneumoniae* genes. These two pathogens were selected as representative Gram-negative and Gram-positive pathogens, as the corresponding host responses show a high degree of separation in principal component analysis (PCA). The Markov clustering algorithm (MCL) was used with an inflation parameter of 4.0, and networks with connected nodes are shown based on high confidence scores (>0.7). STRING analysis was based on *E. coli* str. K-12 substr. MG1655 and *St. pneumoniae* strain ATCC BAA-255/R6.

### Statistical analysis

Statistical differences between RNA extraction protocols were assessed in GraphPad Prism (v10.0.0, GraphPad Software). The Shapiro–Wilk test was used to determine the normality of the residuals (*a*=0.05). Unpaired two-tailed t-tests were used for the comparison of two datasets. If the normality assumption was not met, a non-parametric Mann–Whitney test was used to compare datasets. *P* values: <0.05 (*), 0.002 (**), <0.001 (***), <0.0001 (****).

## RESULTS

### Dual-species RNA extraction of host and pathogen transcripts

**Fig. 1.**
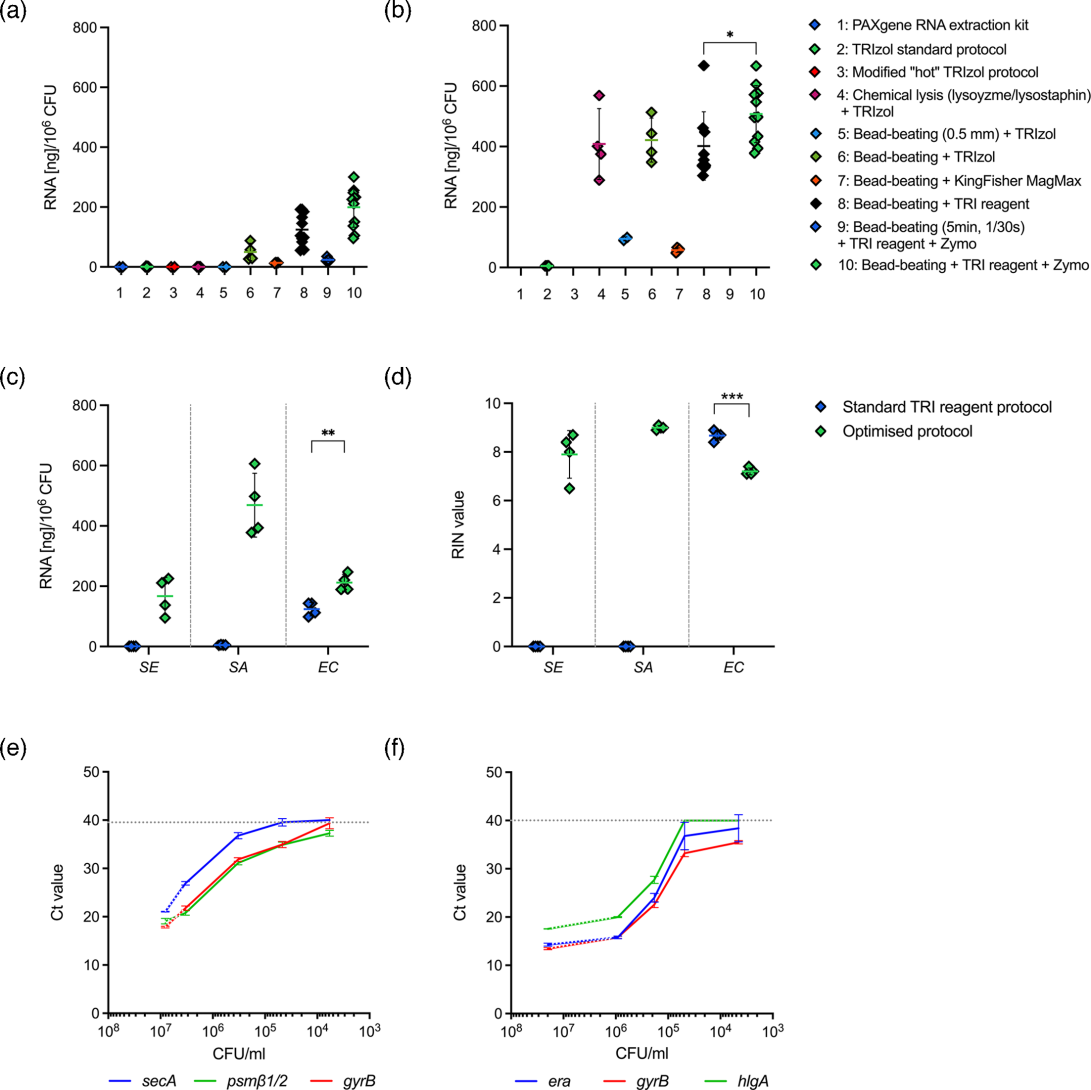
Total (a) *S. epidermidis* and (b) *S. aureus* RNA yield (ng per 10^6^ c.f.u). across different RNA extraction protocols tested. Bead-beating was performed using either the Mini-Beadbeater24 (Bertin) or TissueLyser (Qiagen) with 0.1 or 0.5 mm silica/zirconia beads. Detailed protocols for all tested RNA extraction protocols can be found in File S1. Comparison of our optimized extraction protocol and the unmodfied manufacturer’s protocol (TRI reagent^™^) for RNA isolation from *S. epidermidis* (SE), *S. aureus* (SA) and *E. coli* (EC) stocks in terms of (c) RNA yield (ng per 10^6^ c.f.u) and (d) RNA integrity. Integrity of purified RNA is shown as RIN values (0-10) measured by Agilent TapeStation. Targeted RT-qPCR of (e) *S. epidermidis* and (f) *S. aureus* housekeeping (*secA, gyrB, era*) and virulence (*psmb1/2, hlgA*) genes in purified bacterial RNA samples extracted from decreasing bacterial doses (c.f.u. ml^−1^). The highest bacterial dose (~10^7^ c.f.u. ml^−1^) was included from a separate experiment (dotted line) for comparison. RT-qPCR cut-off set at a *Ct* value of 40. All data are shown as mean±sd. Statistical differences between protocols were determined using t-tests.

Prior to measuring bacterial RNA transcript recovery from whole blood, we first established a baseline for maximum *S. epidermidis* and *S. aureus* RNA recovery (ng per 10^6^ c.f.u.) from pure bacterial stocks. In a preliminary optimization experiment, a range of RNA extraction kits and protocols were trialled, including both chemical and mechanical cell lysis methods (all protocols are detailed in File S1). The highest RNA yields from pure cultures of *S. epidermidis* and *S. aureus* were obtained using mechanical bead-beating in combination with phenol-based extraction (TRI reagent^™^), followed by column-based RNA elution and concentration using a commercial kit (Zymo) (Fig. 1a, b).

To assess the protocol’s applicability to other sepsis-causing bacteria, we compared the total RNA yield and RNA quality (RNA integrity number, RIN) obtained with our optimized protocol to the unmodified TRI reagent^™^ protocol with *E. coli* 29523 as a Gram-negative representative. RNA was readily extracted from *E. coli* using the standard TRI reagent^™^ protocol without additional mechanical lysis (Fig. 1c). Although the optimized protocol yielded higher total *E. coli* RNA (ng) per million c.f.u. (211.8±27.8 vs. 124.2±22.4, respectively; *P*=0.03), the RNA transcripts showed reduced integrity when bead-beating was included (7.2±0.1 vs. 8.7±0.2, respectively; *P*<0.001) (Fig. 1d).

A 10-fold titration series of pure *S. epidermidis* and *S. aureus* stocks was used to determine the sensitivity of the optimized protocol. Targeted RT-qPCR was employed to measure the expression of selected bacterial housekeeping and virulence genes, establishing the lower limit of detection for staphylococcal RNA transcripts (Fig. 1e, f).

**Fig. 2.**
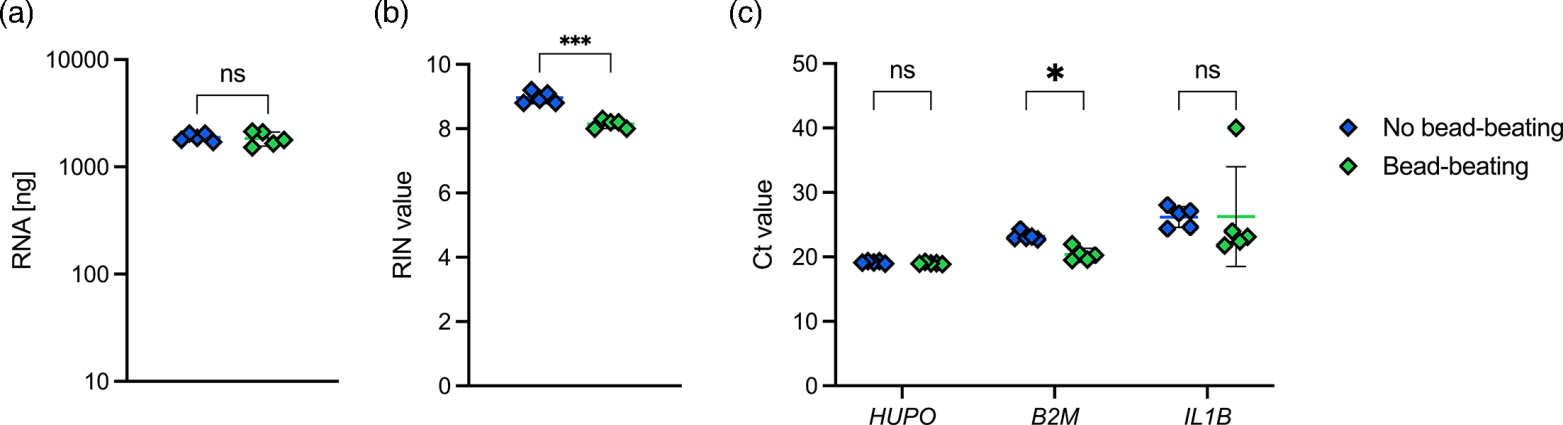
(a) Total host RNA (ng) in 0.5 ml blood was measured using Qubit. (b) RNA quality (RIN; 0-10) was measured using Agilent TapeStation. (c) Targeted RT-qPCR of human genes (*HUPO*, *B2M*, *IL1B*). RT-qPCR cut-off set at a *Ct* value of 40. A single blood donor sample was used for this experiment. Replicate samples are shown as mean±sd (*n*=5). Unpaired two-tailed t-tests (RNA yield and RNA integrity) and Mann–Whitney test (Human gene expression) were used to determine statistical differences between protocols.

To assess the effect that bead-beating has on host leukocyte RNA yield and integrity, we collected healthy adult blood samples (0.5 ml) into PAXgene^™^ Blood RNA solution and purified total RNA from washed blood leukocytes using the optimized bacterial RNA extraction protocol, with and without performing mechanical lysis. Both protocols yielded similar amounts of total host RNA (*P*=0.732) (Fig. 2a). However, significantly lower RNA integrity numbers (RINs) were observed in samples subjected to bead-beating compared to no bead-beating (7.2±0.1 vs. 8.7±0.2, respectively; *P*<0.001) (Fig. 2b). This appeared to be due to fragmentation of the largest host rRNA species (28S rRNA, ~5 kb) during mechanical cell lysis (Fig. S2). There were no significant differences in the expression of human house-keeping gene *HUPO* and host defence gene *IL1B* between samples which did and did not undergo bead-beating as measured by targeted RT-qPCR (Fig. 2c). However, the expression of housekeeping gene *B2M* was significantly lower in the samples where bead-beating was applied (*Ct*=23.2±0.6 vs. 20.4±1.0, respectively; *P*=0.0079).

Clinical blood samples for transcriptomic analysis are typically collected into RNA-stabilizing reagents like PAXgene^™^ Blood RNA Solution, which allows sample storage and transport at various temperatures. However, to the best of our knowledge, the impact of sample storage in PAXgene^™^ on bacterial RNA stability has not yet been determined. To ensure our protocol’s compatibility with clinical sample handling, we inoculated 0.5 ml blood samples with a defined dose of *S. epidermidis* or *S. aureus* (~10^7^ c.f.u. ml^−1^). Samples were processed either after 2 h of incubation in PAXgene*™* at room temperature or after 7 days of storage at −80 °C. RT-qPCR analysis indicated that short-term frozen storage did not affect the expression of selected host and bacterial genes compared to immediately processed samples (Fig. S3).

### Dual RNA-seq of clinical sepsis samples

To validate our optimized DRIB protocol for dual RNA-seq in clinical sepsis samples, we conducted a pilot study using eight sepsis samples from adult patients, collected between September 2021 and January 2023, all with confirmed positive blood cultures (BCs) (Table 1).

**Table 1.** Description of clinical samples (*n*=8) collected for dual RNA-seq in this study.Causative sepsis pathogen was determined via routine BC analysis. Total RNA yield was determined by Qubit. RIN values were determined by Agilent TapeStation. BC=blood culture.

	Sample collection date	BC time-to-positivity (h:min)	Organism (Gram stain, −/+)	Total RNA yield (μg)	RIN
**1**	27-09-2021	11:16	*Escherichia coli* (−)	5.39	8.3
**2**	12-10-2021	16:56	*Staphyolococcus epidermidis* (+)	5.64	8.5
**3**	09-11-2021	17:52	*Serratia marcescens* (−)	3.34	8.4
**4**	19-07-2022	06:24	*Streptococcus pneumoniae* (+)	2.19	7.7
**5**	17-08-2022	09:20	*Streptococcus pneumoniae* (+)	4.90	8.1
**6**	20-10-2022	11:26	*Staphylcoccus aureus* (+)	6.91	8.3
**7**	07-11-2022	10:54	*Streptococcus dysgalactiae* (+)	3.95	7.7
**8**	03-01-2023	13:25	*K. pneumoniae* (−)	5.18	7.9

BC, blood culture.

Blood samples (0.5 ml) were collected into EDTA tubes and stabilized in PAXgene^™^ Blood RNA Solution before our DRIB protocol was used to isolate total host and pathogen RNA. Purified RNA samples were of high quality (RIN: 7.7–8.5) and subjected to deep sequencing using the NovaSeq 6000 Illumina platform. Of the total sequencing reads per sample, 11.8–18.6 million reads were aligned to either the host or bacterial pathogen genomes (Table 2). After read filtering, 92.1–99.1% of the uniquely aligned reads mapped to the human genome, while 0.9–7.9% mapped to the pathogen genomes.

**Table 2.** Number of reads in each sample which were aligned, multi-mapping or unmapped,Aligned reads were filtered and low-count genes were removed. The proportion of uniquely aligned and filtered host and bacterial reads is shown.

	Sequencing reads per sample	Total aligned reads	Filtered aligned reads				Multi-mapped reads		Short reads/ unmapped
			*Host*	*%*	*Bacteria*	*%*	*Host*	*Bacteria*	
**1**	38,200,183	14,712,207	**8,355,631**	56.8	**79,496**	0.5	2,318,668	1,679,174	19,490,134
**2**	39,478,642	13,696,344	**9,225,616**	67.4	**645,268**	4.7	2,312,492	109,676	23,360,130
**3**	39,406,394	17,879,196	**10,570,030**	59.1	**690,488**	3.9	2,388,291	105,766	19,033,141
**4**	46,477,667	18,646,375	**10,887,795**	58.4	**319,297**	1.7	2,246,267	1,580,598	24,004,427
**5**	39,436,489	15,312,160	**8,614,269**	56.3	**132,695**	0.9	2,185,567	113,132	21,825,630
**6**	43,839,143	11,870,509	**8,025,505**	67.6	**789,808**	6.7	3,601,973	1,109,299	27,257,362
**7**	47,957,251	18,143,533	**9,250,408**	51.0	**738,044**	4.1	2,444,690	111,665	27,257,363
**8**	39,653,645	14,656,468	**9,344,100**	63.8	**567,002**	3.9	2,181,058	95,575	22,720,544

### Host-specific transcriptional responses during sepsis

**Fig. 3.**
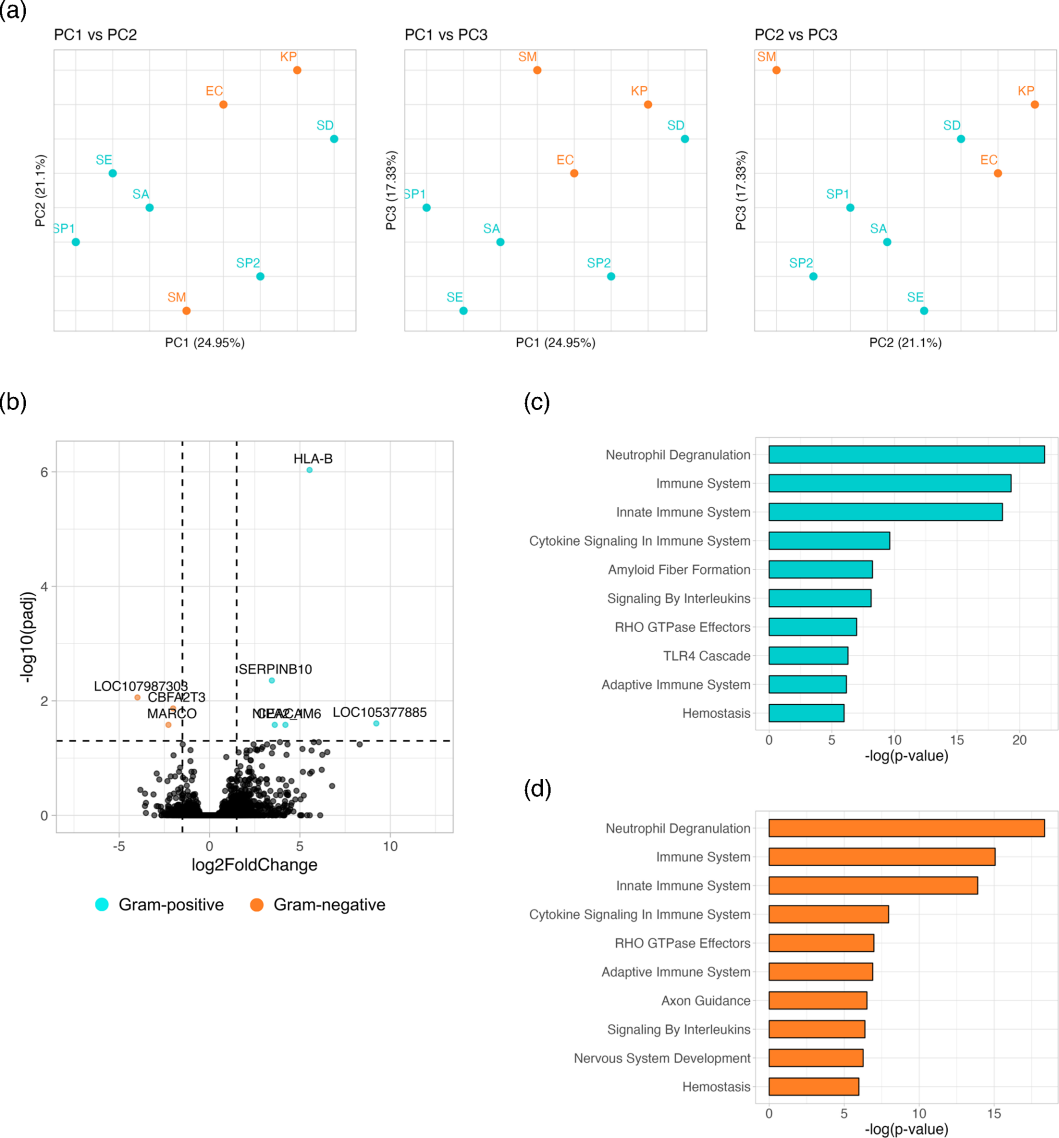
(a) PCA was performed on the 100 most highly expressed host genes. (b) Volcano plot showing DEG analysis of host genes across clinical sepsis samples identified as significant if Benjamini–Hochberg adjusted *P* values were <0.05 and log FC were >2 using the DESeq2 package in R. Reactome pathway analysis based on the 100 most highly expressed host genes across (c) Gram-positive and (d) Gram-negative sepsis samples.

Following data filtering and normalization, we performed exploratory analyses to identify differential host responses between patients with sepsis caused by Gram-positive or Gram-negative pathogens. Principal Component Analysis (PCA) was used to reduce the dimensionality of host gene expression and uncover significant patterns and variations. PCA showed that the causative pathogen (Gram-positive or Gram-negative) did not constitute a main source of variation in the 100 most highly expressed host genes, with PC1 (24.95%) and PC2 (21.1%) accounting for <50% of variability (Fig. 3a).

Differential gene expression (DEG) analysis moreover indicated few significant differences in host in gene expression between sepsis caused by Gram-positive and Gram-negative bacteria in this pilot cohort (Fig. 3b). Five host genes were significantly upregulated (adjusted *P*<0.05, FC>2) in Gram-positive sepsis samples (*HLA-B*, *SERPINB10*, *LOC105377885*, *CEACAM6*, *NIPA2*), while three genes were upregulated in Gram-negative sepsis samples (*CBFA2T3*, *LOC107987303*, *MARCO*).

We used Reactome pathway analysis to identify enriched host pathways in the top 100 most highly expressed genes and found that both Gram-positive and Gram-negative sepsis samples exhibited significant enrichment (*P* and *Q* value<0.001) in host innate immune responses, including pathways related to neutrophil degranulation, innate immune signalling and cytokine signalling (Fig. 3c, d).

To identify host gene expression signatures characteristic of sepsis, in the absence of pre- or post-sepsis baseline samples from these patients, we measured the unstimulated transcriptional responses from three healthy adult blood samples and conducted transcriptional profiling using DEG analysis, PCA and Weighted Gene Co-expression Network Analysis (WGCNA).

**Fig. 4.**
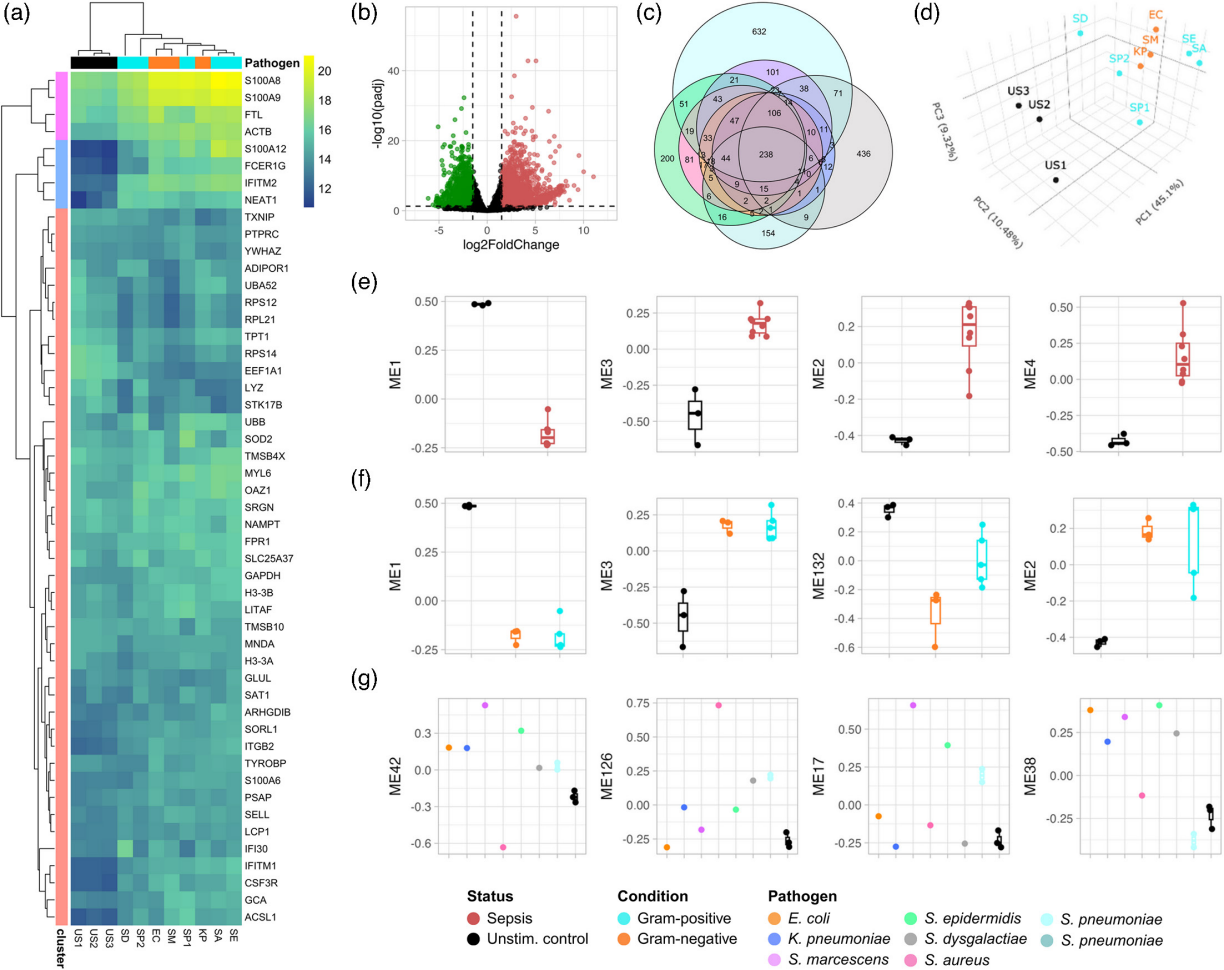
(a) Heatmap showing the top 50 expressed host genes in healthy and sepsis samples. Gene expression data were normalized using VST using the DESeq2 R package, and *k*-means and Euclidean clustering were performed. (b) Volcano plot showing DEGs in sepsis samples compared to healthy controls (log2FC>2, BH adjusted *P*>0.05). (c) Venn-Euler diagram showing pathogen-specific DEG lists. (d) 3D PCA. (e)–(g) Four top-ranked MEs identified by WGCNA with differential expression across clinical traits/conditions.

*K*-means and hierarchical (Euclidean) clustering were performed on the 50 most highly expressed genes in sepsis and healthy samples. The resulting heatmap revealed a cluster of genes (cluster 2) with markedly higher expression in sepsis samples compared to healthy samples, including S100 calcium-binding protein A12 (*S100A12*), interferon-induced transmembrane protein 2 (*IFITM2*), nuclear enriched abundant transcript 1 (*NEAT1*) and Fc epsilon receptor Ig (*FCER1G*) (Fig. 4a). Despite a broad similarity in the overall gene expression profiles indicating common host response mechanisms during sepsis, we also observed distinct patterns within and between Gram-positive and Gram-negative sepsis samples among the most highly expressed genes.

DEG analysis identified 2,484 genes differentially expressed (>2 fold change, FC; *P*<0.05) between sepsis and healthy controls, with 1,543 upregulated and 941 downregulated genes (Fig. 4b). Among these, only 169 differentially expressed genes (DEGs) were consistently present across all sepsis samples, irrespective of the causative pathogen. A significant number of DEGs were unique to individual sepsis samples, as depicted in the Venn-Euler diagram showing pathogen-specific host DEGs overlap (Fig. 4c).PCA further highlighted the distinct transcriptional landscape of sepsis samples compared to unstimulated controls (Fig. 4d).

WGCNA identified gene modules (module eigengenes, MEs) which were significantly differentially co-expressed in samples with different disease status (healthy vs sepsis), pathogen type (Gram-negative vs Gram-positive) and individual (pathogen-specific) profiles (Fig. 4e–g). For instance, gene module ME1 was downregulated, while ME2, ME3 and ME4 were upregulated in sepsis samples compared to healthy controls (Fig. 4e). ME2, which comprises 1,679 genes, was found enriched in genes with roles in immune and cellular pathways, including leukocyte and natural killer (NKcell-mediated immunity, IL-6 production and Fc receptor signalling. While Gram-positive and Gram-negative samples shared similar gene co-expression profiles, more variability was observed among Gram-positive samples (Fig. 4f). Distinct expression patterns were also seen when comparing individual sepsis samples, with gene modules ME42, 126, 17 and 38 showing the most significant differences in co-expression (Fig. 4g).

### Bacterial transcriptional responses during sepsis

**Fig. 5.**
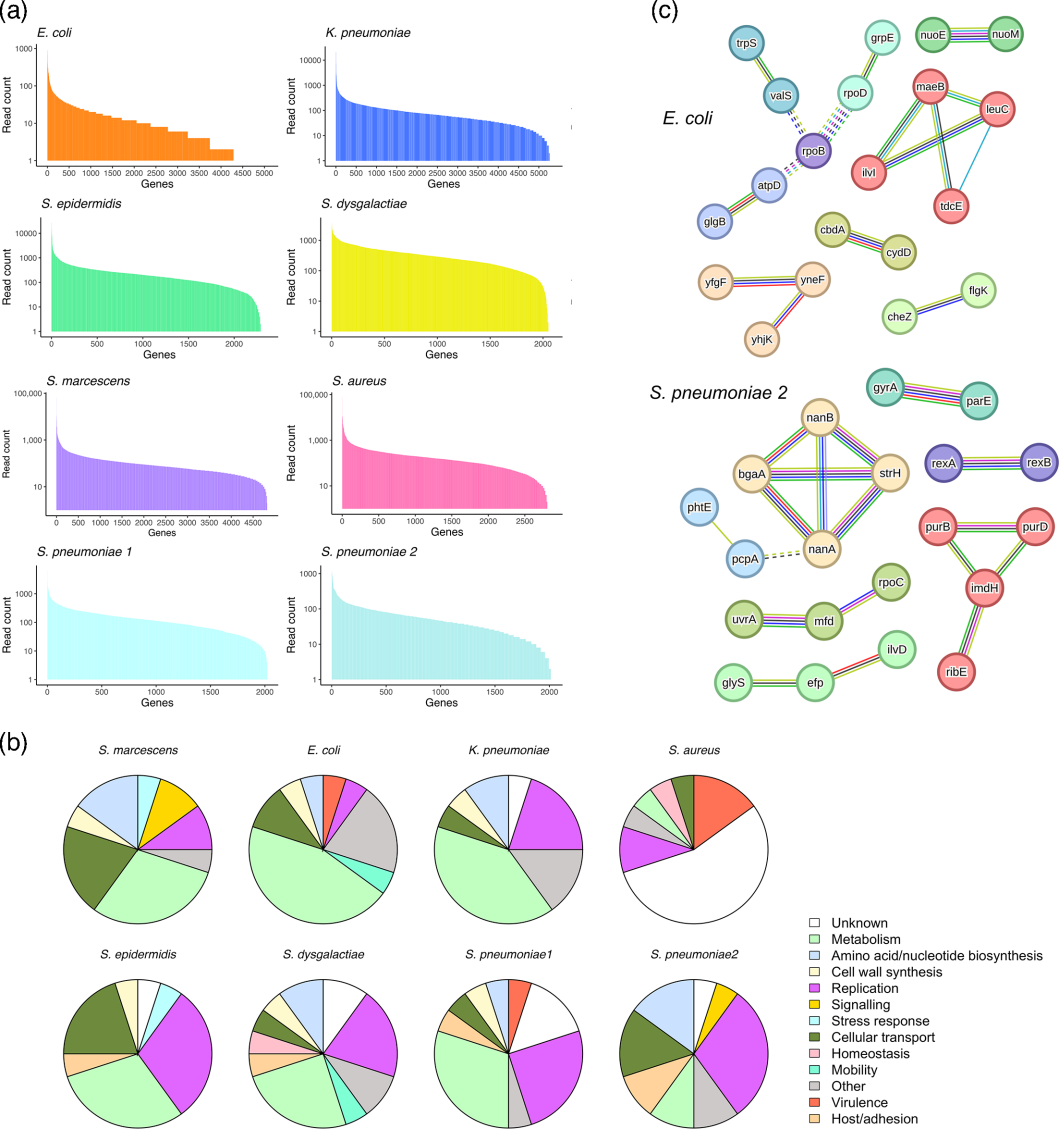
(a) Sequencing coverage of genomic bacterial references in clinical samples. Aligned bacterial reads were sorted and are shown with decreasing counts across the total number of gene sequences included in each bacterial reference. (b) Pie charts showing the distribution of functional categories across the top 20 most highly expressed bacterial genes in sepsis samples. Genes were assigned to categories by manual gene annotation based on databases/literature. (c) Full STRING protein interaction networks of top 100 *E. coli* and *St. pneumoniae* genes. The minimum required interaction score was set to 0.7 (high confidence). Edges between individual MCL clusters are depicted as dotted lines. Unconnected nodes were removed from the networks.

We then analysed the reads which aligned to bacterial reference genomes in each sepsis sample. We observed a high coverage across the distinct bacterial genomic sequences included in our bacterial references for all species (Fig. 5a). Notably, certain bacterial genes, such as *S. aureus* fibronectin-binding protein B (*SAOUHSC_02802*) and *Se. marcescens* YcfL family protein (*C7M65_RS13175*), exhibited particularly high expression levels, with 71,248 and 62,576 read counts, respectively (Tables S1 and S3).

To understand bacterial gene expression during sepsis, the 20 most highly expressed genes from each pathogen were manually annotated and categorized into functional groups, including metabolism, amino acid/nucleotide biosynthesis, cell wall synthesis, replication, signalling, stress response, host-interaction/adhesion and potential virulence factors. Bacterial metabolism genes were among the most prevalent genes expressed, particularly in Gram-negative pathogens (Fig. 5b). In contrast, genes which are involved in bacterial replication were seen at higher abundances among the most highly expressed genes in Gram-positive pathogens. Our analysis was limited by incomplete genome annotations, resulting in a significant number of genes categorized as encoding for ‘hypothetical proteins’. Among the characterized *S. aureus* genes, several were identified as having potential roles in virulence, such as fibronectin-binding protein B and clumping factor genes. *St. pneumoniae* also expressed genes encoding for proteins associated with host adhesion and bacterial virulence, such as LPXTG-anchored adhesin/beta-galactosidase (*BgaA*), isopeptide-forming domain-containing fimbrial protein (*SPNHU17_RS02450*) and choline-binding protein (*PcpA*).

To further explore bacterial gene expression during infection, we constructed a protein–protein interaction network using STRING for the top 100 highly expressed genes from a representative Gram-negative species (*E. coli*) and Gram-positive species (*St. pneumoniae*, sample 1). This analysis mapped 72 *E. coli* genes and 38 *St. pneumoniae* genes to proteins, identifying 58 and 68 protein–protein associations, respectively. MCL clustering revealed nine protein clusters in *E. coli*, with RNA polymerase beta subunit (rpoB) as a central node for three clusters, including those involved in tRNA synthesis, ATP synthase and respiratory chain enzymes (Fig. 5c). Other clusters included cyclic-di-GMP synthesis and bacterial chemotaxis-related genes. In *St. pneumoniae*, seven protein clusters centering around genes involved in chromosome organization and nucleotide metabolism were identified, with metal-ion binding being an enriched molecular function.

## Discussion

Advances in RNA-seq have revealed complex host transcriptional responses to sepsis and highlighted the heterogeneous nature of sepsis across host populations and infectious aetiologies [41,45]. However, the role of host–pathogen interactions in sepsis onset and outcomes is less well understood. Our study aimed to fill this gap by analysing bacterial gene expression in blood samples from sepsis patients alongside host transcriptional responses, using an optimized dual-species RNA isolation protocol. A pilot set of clinical sepsis samples was then used to validate this protocol with a dual RNA-seq approach and explore patient and bacterial transcriptional patterns.

By combining our optimized “DRIB” protocol with dual-species rRNA depletion and deep sequencing, we were able to map between 0.94 and 7.92% (79,496–789,808 reads) of total aligned and filtered sample reads to bacterial references in our pilot sample cohort. Due to gene database limitations, some non-bacterial sequences were present in the *E. coli* and *St. pneumoniae* reference files. Reads mapped to these sequences were removed, which likely reduced the total bacterial reads for *E. coli* samples.

Only a small number of host genes were differentially expressed between the Gram-positive (*HLA-B*, *SERPINB10*, *LOC105377885*, *CEACAM6*, *NIPA2*) and Gram-negative (*CBFA2T3*, *LOC107987303*, *MARCO*) sepsis samples in our cohort. While some of these genes have been associated with human diseases [46,50], their roles in sepsis pathogenesis remain unclear. Our findings align with previous studies reporting a largely conserved transcriptional host response in sepsis [51], with significant enrichment in innate immune responses, including pathways related to neutrophil degranulation, innate immune signalling and cytokine signalling. While our findings suggest that pathways related to nervous system development and metal binding processes may be upregulated specifically in Gram-negative sepsis, the small sample size in this study and significant potential confounding factors do not allow for definitive conclusions.

Comparative analysis with healthy controls revealed both conserved and pathogen-specific host DEGs. Among these, we found certain S100 family proteins (i.e. S100A12 and S100A11) exclusively elevated in sepsis samples, while traditional pro-inflammatory S100A8 and S100A9 were similarly expressed in both healthy and sepsis samples. Differential expression of S100 proteins may be indicative of their varying contributions to the immune system’s response to homeostatic challenges and pathogenic threats [52]. WGCNA highlighted gene modules differentially expressed in sepsis and healthy samples, Gram-positive and Gram-negative samples, as well as individual sepsis samples. These findings indicate that specific gene networks (gene modules) are differentially regulated depending on the individual host immune characteristics, the type of sepsis-inducing pathogen, and likely also infectious origin and sepsis timepoint.

We found a high coverage of bacterial gene sequences (>2,000 mapped genes) in the sepsis samples across all patients, with elevated counts of a select number of highly expressed bacterial genes. Genes related to bacterial metabolism, cellular transport and replication were consistently among the most expressed genes across all sepsis-causing bacterial species. Gram-negative pathogens expressed a higher proportion of genes associated with metabolic functions, suggesting enhanced adaptation to host environmental pressures [53]. In contrast, Gram-positive pathogen gene experssion appears to focus increasingly on replication and cell wall synthesis. This may indicate strategies for increased growth and resistance to host defences [54,55], potentially influenced by the selective pressures exerted by empiric antibiotic therapy. Protein network analyses of the top 100 genes expressed by *E. coli* and *St. pneumoniae* identified bacterial genes involved in vital processes such as DNA and nucleic acid metabolism, tRNA synthesis and respiratory chain functions as highly induced. Similar bacterial gene expression patterns were observed in previous microarray studies when pathogens such as *S. aureus*, *Neisseria meningitidis*, *Enterococcus faecalis* and *Streptococcus agalactiae* were exposed to human blood or serum [56,59], suggesting a conserved bacterial strategy in response to the hostile host environment. However, this pilot study included only one or two samples per bacterial pathogen, and functional categories were assigned based on assumed/known functions. Further research using larger sample sizes is needed to confirm these findings.

## Limitations

This pilot study aimed to assess the feasibility of dual RNA-seq for sepsis research using an optimized protocol for host and bacterial RNA recovery from clinical sepsis blood samples. Our small sample size limits the ability to draw firm conclusions about the biological mechanisms of sepsis. Future studies in larger cohorts could employ targeted diagnostic methods, such as PCR, to accurately identify all causative pathogens (i.e. polymicrobial sepsis, which is often underreported [60]) and determine bacterial blood burdens in sepsis samples. Additionally, our analysis could not account for confounding factors such as patient demographics or sepsis origin. Future studies may include samples of non-sepsis causes of inflammation as additional control groups to determine host immune responses specific to sepsis. Patients were selected based on BC-positive sepsis, with pathogens identified at the species level. To enhance bacterial read mapping, we compiled a pan-genome reference from NCBI’s gene sequences for each bacterial genome. The use of whole-genome bacterial references, obtained through whole-genome sequencing of causative clinical strains, would likely further improve accurate bacterial read alignment.

## Conclusions

This study presents a novel approach utilizing dual RNA-seq to investigate host and bacterial gene expression during sepsis, marking a significant advancement over existing approaches that often focus on one or the other. By employing our optimized DRIB protocol, we successfully extracted high-quality RNA from low-volume (0.5 ml) clinical sepsis samples, capturing transcriptional data from both host and their diverse bacterial pathogens. Key findings include the observation that sepsis induces common host immune-metabolic pathways, largely independent of the causative pathogen (Gram-positive or Gram-negative). In parallel, the study highlighted the heterogeneity of individual host responses, which might be driven by both host- and pathogen-specific factors as well as disease status and origin. Lastly, the enriched host and bacterial pathways suggest host–pathogen interactions, particularly in metal acquisition and bacterial host adhesion. Despite the limited number of samples, which constrains the statistical power of expression-based analyses, this study demonstrates the robustness and potential of the proposed protocol. It lays the groundwork for future research aimed at exploring the complexities of host–pathogen interactions in sepsis, offering an improved methodology that could enhance our understanding of disease mechanisms.

## Supplementary material

10.1099/mgen.0.001501 Uncited Supplementary Material 1.

## Abbreviations

BC: blood culture
DEGs: differentially expressed host genes
DRIB: dual RNA isolation from blood
dual RNA-seq: dual RNA-sequencing
FC: fold change
HBA: horse blood agar
MCL: Markov clustering algorithm
MEs: module eigengenes
mid-log: mid-logarithmic
NCBI: National Center for Biotechnology Information
NFW: nuclease-free water
PCA: principal component analysis
RIN: RNA integrity number
RT-qPCR: reverse transcription-quantitative PCR
STRING: Search Tool for the Retrieval of Interacting Genes
TSB: trypticase soy broth
VST: variance-stabilizing transformation
WGCNA: weighted gene co-expression network analysis
