# Supplementary material for: Dual RNA isolation from blood: an optimized protocol for host and bacterial RNA purification for dual RNA-sequencing analysis in whole blood sepsis samples
Source: Microb Genom. 2025 Sep 18;11(9):001501. doi: 10.1099/mgen.0.001501 (PMC12447629; doi:10.1099/mgen.0.001501)
Supplement: Uncited Supplementary Material 1. [file mgen-11-01501-s001.pdf]

Dual RNA isolation from blood: an optimised protocol for host and bacterial RNA purification for dual RNA-sequencing analysis in whole blood sepsis samples

Isabella Anna Joubert, Christopher Mullally, Edward Litton, Edward Raby, Abha Chopra, Tobias Strunk, Penghao Wang, Andrew Currie* (a.currie@murdoch.edu.au)

Supplementary

<u>Content</u>

Results:

Supplementary Figure 1: Schematic of optimised DRIB protocol for host and pathogen transcript isolation from low-volume (0.5 ml) blood samples.

Supplementary Figure 2: Total host RNA in 0.5 ml PAXgeneTM-stabilised blood was extracted using the DRIB protocol with and without the inclusion of mechanical lysis (bead-beating).

Supplementary Figure 3: Effect of short-term frozen storage (-80°C) of PAXgene$^{\text{TM-}}$stabilised samples.

Supplementary Table 1: Top 20 *S. marcescens* and *S. dysgalactiae* genes in clinical sepsis samples with gene name and gene symbol (where available) or gene ID

Supplementary Table 2: Top 20 *E. coli* and *K. pneumoniae* genes in clinical sepsis samples with gene name and gene symbol (where available) or gene ID.

Supplementary Table 3: Top 20 *S. epidermidis* and *S. aureus* genes in clinical sepsis samples with gene name and gene symbol (where available) or gene ID.

Supplementary Table 4: Top 20 *S. pneumoniae* in clinical sepsis samples with gene name and gene symbol (where available) or gene ID.

Material and Methods

Bacterial lysis

Column-based RNA extraction

Guanidine thiocyanate/phenol-based RNA extraction:

RNA clean-up and concentration

Supplementary Table 5: Molecular characteristics of in-house designed bacterial and host primers and Multiplex probes. FWD: Forward primer; REV: Reverse primer; bp: base pairs.

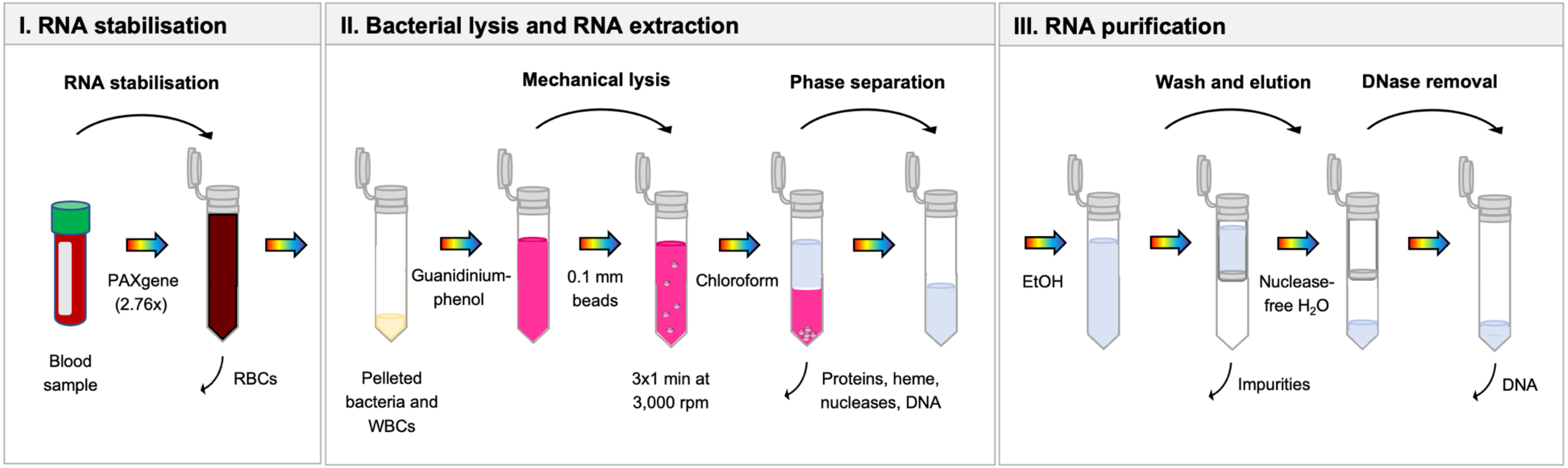

Supplementary Figure 1: Schematic of optimised DRIB protocol for host and pathogen transcript isolation from low-volume (0.5 ml) blood samples

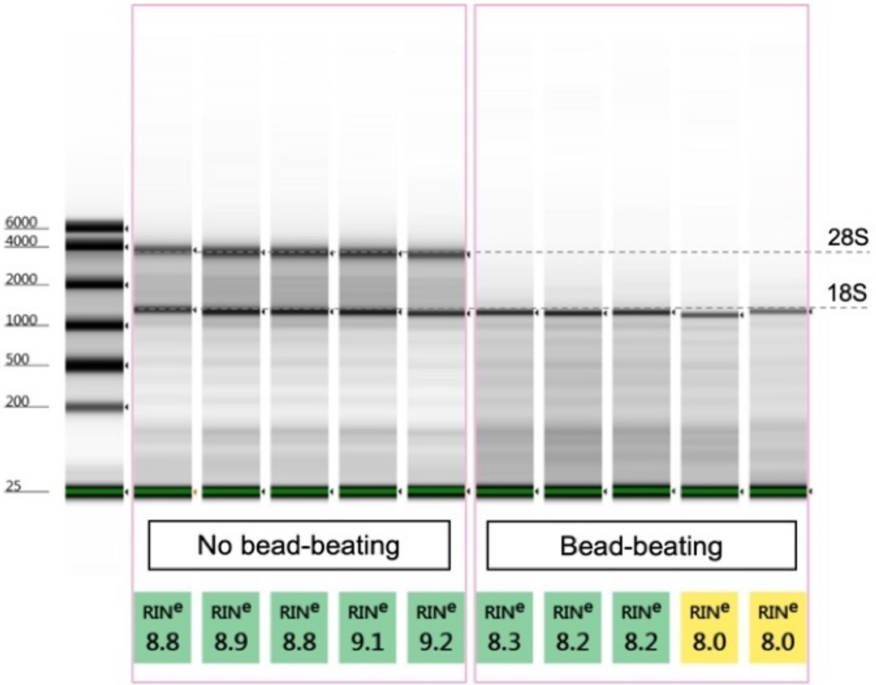

Supplementary Figure 2: Total host RNA in 0.5 ml PAXgeneTM-stabilised blood was extracted using the DRIB protocol with and without the inclusion of mechanical lysis (bead-beating). RNA quality of purified host RNA was measured using Agilent TapeStation and gel images are shown, indicating the absence of the human 28S rRNA peak in samples that underwent bead-beating.

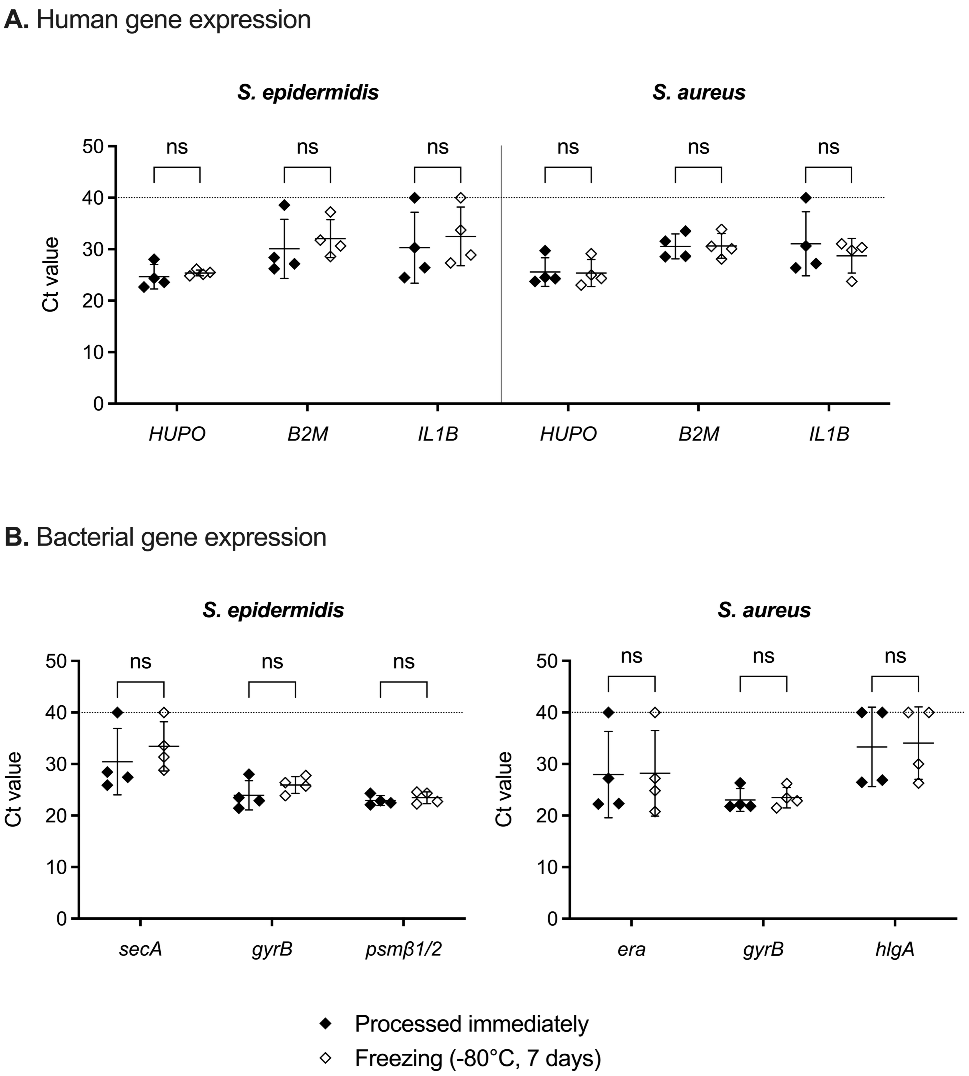

Supplementary Figure 3: Effect of short-term frozen storage (-80°C) of PAXgene^{TM-}stabilised samples. **A.** Host and **B.** Bacterial gene expression measured via targeted RT-qPCR. 0.5 ml whole blood samples were inoculated with ~10^7 CFU/ml of *S. epidermidis* or *S. aureus* and stabilised with 1.38 ml PAXgene^{TM}. Samples were then either processed after 2h incubation at RT or stored at -80°C for 7 days and then processed. Replicate measurements are shown as mean ± SD (n=4). Mann-Whitney test (A, B-SE) and unpaired two-tailed t-test (B-SA) were used to determine statistical differences between processing protocols.

Supplementary Table 1: Top 20 *S. marcescens* and *S. dysgalactiae* genes in clinical sepsis samples with gene name and gene symbol (where available) or gene ID.

S. marcescens	***Count***	***S. dysgalactiae***	***Count***
YcfL family protein (C7M65_RS13175)	*62,576*	Thymidylate synthase (I6H74_RS05820)	*3,712*
Glycosyltransferase family A protein (C7M65_RS08175)	*11,048*	Hypothetical protein[1] (I6H74_RS00635)	*3,190*
Diaminopimelate decarboxylase (lysA)	*9,002*	Redoxin NrdH (I6H74_RS08385)	*2,694*
HAMP domain-containing sensor histidine kinase (C7M65_RS03780)	*6,982*	UDP-N-acetylmuramoyl-L-alanine--D-glutamate ligase (murD)	*2,678*
Nucleoid-associated protein YejK(yejK)	*6,240*	SNF2-related protein (I6H74_RS05970)	*2,462*
MFS transporter (C7M65_RS06365)	*4,014*	Thiolase family protein (I6H74_RS04310)	*2,202*
GntP family transporter (C7M65_RS15500)	*3,792*	Glycerol-3-phosphate 1-O-acyltransferase PlsY (plsy)	*2,166*
FMNH2-dependent alkanesulfonate monooxygenase (ssuD)	*3,690*	MarC family protein (I6H74_RS01810)	*2,146*
Pirin family protein (C7M65_RS01070)	*3,610*	Pilin N-terminal domain-containing protein (I6H74_RS00810)	*2,104*
Bcr/CflA family multidrug efflux MFS transporter (C7M65_RS05850)	*3,386*	NEAT domain-containing protein (I6H74_RS03385)	*1,974*
DNA-directed RNA polymerase subunit beta (rpoC)	*3,234*	PolC-type DNA polymerase III I6H74_RS00340)	*1,794*
DMT family transporter (C7M65_RS02155)	*2,248*	Type II CRISPR RNA-guided endonuclease Cas9 (cas9)	*1,762*
Cystine ABC transporter substrate-binding protein (tcyJ)	*2,204*	Helix-turn-helix transcriptional regulator (I6H74_RS01845)	*1,730*
Ornithine decarboxylase SpeF (speF)	*2,182*	S8 family serine peptidase (I6H74_RS10125)	*1,718*
Methyltransferase (C7M65_RS17445)	*2,176*	DUF3173 family protein (I6H74_RS00535)	*1,714*
LysR family transcriptional regulator (C7M65_RS04615)	*1,958*	Bifunctional acetaldehyde-CoA/alcohol dehydrogenase (adhE)	*1,696*
Glycosyl hydrolase family 18 protein (C7M65_RS22235)	*1,826*	GbpC/Spa domain-containing protein (I6H74_RS05955)	*1,660*
Inositol-1-monophosphatase (suhB)	*1,816*	Bifunctional 2',3'-cyclic-nucleotide 2'-phosphodiesterase/3'-nucleotidase (I6H74_RS00420)	*1,652*
Phenylacetate-CoA oxygenase subunit PaaC (paaC)	*1,774*	Sulfatase-like hydrolase/transferase (I6H74_RS05445)	*1,640*
Acyltransferase family protein (C7M65_RS08825)	*1,706*	SEC10/PgrA surface exclusion domain-containing protein (I6H74_RS03005)	*1,612*

[1]Hypothetical proteins describe proteins predicted to be expressed from an open reading frame (ORF), for which there is no direct experimental evidence of its expression or function.

Supplementary Table 2: Top 20 *E. coli* and *K. pneumoniae* genes in clinical sepsis samples with gene name and gene symbol (where available) or gene ID.

E. coli	***Count***	***K. pneumoniae***	***Count***
Nitrate reductase 1 alpha subunit (narG)	*3,552*	Dihydrolipoamide dehydrogenase (KPHS_09950)	*21,330*
Transketolase (tktB_2)	*2,294*	Sugar isomerase (SIS) (KPHS_05070)	*20,242*
Zn-dependent NAD(P)-binding oxidoreductase (ydjJ)	*1,084*	Diaminopimelate decarboxylase (KPHS_43170)	*7,654*
Inner membrane protein (ybcI)	*1,084*	Head completion/stabilization protein (GpL) (KPHS_17240)	*6,812*
Arabinose efflux transporter (ydeA)	*936*	Lysyl-tRNA synthetase (KPHS_43860)	*3,702*
Lytic murein transglycosylase (slt)	*914*	Cell division protein ZipA (KPHS_38250)	*3,662*
DEAD/DEAH box helicase (ECs_5260)	*848*	Acetyl-CoA carboxylase biotin carboxylase subunit (KPHS_47970)	*3,218*
Leucyl/phenylalanyl-tRNA-protein transferase (aat)	*678*	Hypothetical protein (KPHS_22790)	*3,114*
NADH-quinone oxidoreductase subunit E (nuoE)	*586*	Asparagine synthetase B (KPHS_15280)	*2,740*
Protease IV (sppA)	*562*	DNA-binding transcriptional repressor GlpR (KPHS_49410)	*2,492*
Cytochrome d terminal oxidase subunit I (appC)	*470*	D-alanyl-D-alanine carboxypeptidase (KPHS_35290)	*2,422*
2,3-dihydroxypropane-1-sulfonate exporter (yihP)	*398*	L-serine deaminase 2 (KPHS_42170)	*2,268*
T3SS effector-like protein EspY (espY4)	*392*	Putative negative regulator (KPHS_38180)	*2,242*
Flagellar hook-filament junction protein 1 (flgK)	*366*	Putative dipeptide ABC transport system ATP-binding component (KPHS_35950)	*1,970*
Fe-Mn family superoxide dismutase (sodA)	*330*	Condesin subunit B (KPHS_18320)	*1,878*
RNA helicase (ECs_5263)	*308*	Putative ATP-dependent helicase (KPHS_36830)	*1,762*
D-arabinose 5-phosphate isomerase (kdsD)	*298*	Putative sulfate transporter (KPHS_05380)	*1,506*
23S rRNA m(5)C1962 methyltransferase (rlmI)	*298*	Putative Retron-type reverse transcriptase (KPHS_34960)	*1,454*
Diguanylate cyclase (yneF)	*298*	Putative beta-glucoside kinase (KPHS_38960)	*1,334*
N-acetylneuraminate lyase (nanA)	*298*	Twin arginine translocase protein E(KPHS_14980)	*1,330*

Supplementary Table 3: Top 20 *S. epidermidis* and *S. aureus* genes in clinical sepsis samples with gene name and gene symbol (where available) or gene ID.

S. epidermidis	***Count***	***S. aureus***	***Count***
Ltp family lipoprotein (EQW00_RS07680)	*26,588*	Fibronectin binding protein B (SAOUHSC_02802)	*71,248*
Hyperosmolarity resistance protein Ebh (ebh)	*15,076*	Hypothetical protein (SAOUHSC_01974)	*11,762*
PstS family phosphate ABC transporter substrate-binding protein (EQW00_RS07315)	*11,638*	Hypothetical protein (SAOUHSC_00058)	*11,382*
Ornithine carbamoyltransferase (argF)	*8,660*	Hypothetical protein (SAOUHSC_03024)	*11,116*
DNA topoisomerase III (EQW00_RS03530)	*7,080*	Clumping factor B (SAOUHSC_02963)	*10,448*
Glucosaminidase domain-containing protein (EQW00_RS08885)	*6,258*	Hypothetical protein (SAOUHSC_01253)	*8,352*
Nucleotidyltransferase (EQW00_RS08505)	*4,676*	Coenzyme A disulfide reductase (SAOUHSC_00908)	*7,903*
AAA family ATPase (EQW00_RS05225)	*3,594*	Hypothetical protein (SAOUHSC_02990)	*7,882*
Hypothetical protein (EQW00_RS01435)	*3,430*	Monovalent cation/H+ antiporter subunit D (SAOUHSC_00628)	*5,300*
MobV family relaxase (mobV)	*2,950*	Clumping factor (SAOUHSC_00812)	*5,275*
ABC transporter ATP-binding protein (EQW00_RS12045)	*2,804*	Hypothetical protein (SAOUHSC_01447)	*4,894*
Cell division protein FtsZ (ftsZ)	*2,780*	Hypothetical protein (SAOUHSC_02685)	*4,796*
Fructose-specific PTS transporter subunit EIIC (EQW00_RS01565)	*2,588*	Hypothetical protein (SAOUHSC_00025)	*4,725*
Nitrite reductase large subunit NirB (nirB)	*2,580*	Adenylosuccinate lyase (SAOUHSC_02126)	*4,661*
MFS transporter (EQW00_RS12025)	*2,128*	Amino acid ABC transporter permease (SAOUHSC_00732)	*4,094*
YSIRK signal domain/LPXTG anchor domain surface protein (EQW00_RS05555)	*2,122*	Lytic regulatory protein (SAOUHSC_02390)	*3,871*
PTS sugar transporter subunit IIC (EQW00_RS02275)	*2,120*	DNA-directed RNA polymerase subunit beta (rpoB)	*3,838*
NADH dehydrogenase subunit 5 (EQW00_RS11695)	*2,004*	Hypothetical protein (SAOUHSC_01931)	*3,672*
DNA mismatch repair endonuclease MutL (mutL)	*2,000*	Hypothetical protein (SAOUHSC_00034)	*3,502*
Class 1b ribonucleoside-diphosphate reductase subunit alpha (nrdE)	*1,879*	Hypothetical protein (SAOUHSC_00789)	*3,351*

Supplementary Table 4: Top 20 *S. pneumoniae* in clinical sepsis samples with gene name and gene symbol (where available) or gene ID.

S. pneumoniae 1	***Count***	***S. pneumoniae 2***	***Count***
Phosphoribosylformylglycinamidine synthase (SPNHU17_RS00475)	*6,290*	Glycoside hydrolase family 31 protein (SPNHU17_RS01755)	*1,190*
DUF1836 domain-containing protein (SPNHU17_RS07025)	*2,638*	Phosphoribosylaminoimidazolesuccinocarboxamide synthase (SPNHU17_RS00470)	*1,024*
DNA topoisomerase IV subunit B (parE)	*2,008*	IS3-like element ISSpn4 family transposase (SPNHU17_RS06425)	*1,022*
Methylenetetrahydrofolate reductase [NAD(P)H] (metF)	*1,830*	Phosphoribosylformylglycinamidine synthase (SPNHU17_RS00475)	*980*
Ribosome small subunit-dependent GTPase A (rsgA)	*1,806*	DNA polymerase III subunit alpha (SPNHU17_RS04355)	*976*
Endonuclease MutS2 (SPNHU17_RS02190)	*1,622*	ABC transporter ATP-binding protein (SPNHU17_RS09000)	*862*
Helicase-exonuclease AddAB subunit AddA (addA)	*1,548*	Adenylosuccinate lyase (purB)	*814*
ABC transporter permease subunit Vex3 (vex3)	*1,539*	Hypothetical protein (SPNHU17_RS05470)	*716*
Hypothetical protein (SPNHU17_RS00215)	*1,448*	ABC transporter permease subunit Vex3 (vex3)	*702*
LPXTG-anchored adhesin/beta-galactosidase BgaA (bgaA)	*1,346*	excinuclease ABC subunit UvrA(uvrA)	*610*
UDP-N-acetylmuramoyl-L-alanyl-D-glutamate--L-lysine ligase (SPNHU17_RS07305)	*1,332*	HAMP domain-containing histidine kinase (SPNHU17_RS07760)	*548*
F0F1 ATP synthase subunit alpha (atpA)	*1,317*	S8 family serine peptidase (SPNHU17_RS03175)	*536*
Carbamoyl-phosphate synthase large subunit (carB)	*1,134*	PTS system mannose/fructose/sorbose family transporter subunit IID (SPNHU17_RS10375)	*534*
S8 family serine peptidase (SPNHU17_RS03175)	*1,124*	LPXTG-anchored adhesin/beta-galactosidase BgaA (bgaA)	*510*
Putative DNA binding domain-containing protein (SPNHU17_RS01115)	*1,022*	Endonuclease MutS2 (SPNHU17_RS02190)	*434*
Molecular chaperone DnaJ (dnaJ)	*1,018*	Isopeptide-forming domain-containing fimbrial protein (SPNHU17_RS02450)	*424*
Choline-binding protein PcpA (pcpA)	*960*	M1 family metallopeptidase (SPNHU17_RS03915)	*382*
VOC family protein (SPNHU17_RS00580)	*920*	DNA topoisomerase IV subunit B (parE)	*380*
M1 family metallopeptidase (SPNHU17_RS03915)	*862*	Putative DNA binding domain-containing protein (SPNHU17_RS01115)	*376*
Chromosome segregation protein SMC (smc)	*862*	Chromosome segregation protein SMC (smc)	*374*

Methods

Bacterial lysis

For enzymatic lysis of bacteria before RNA extraction, cryopreserved frozen mid-log stocks were washed twice with PBS. Subsequently, they were incubated with 0.5 ml RNAlater (Thermo Fisher Scientific) at RT for 15 minutes. After a single wash with PBS, lysozyme and lysostaphin were added at a final concentration of 1.5 mg and 20 μg, respectively. Samples were incubated at 37°C on a heating block for either 30 or 60 minutes. Total RNA extraction was performed using 1 ml of TRIzol (Thermo Fisher Scientific) according to the manufacturer's protocol.

Mechanical bacterial lysis was performed by transferring bacterial stocks into 2 ml bead-beating tubes containing zirconia/silica or ceramic beads (BioSpec Products, 0.5 mm; Invitrogen™ PureLink™, 0.1 mm; Bertin Technologies, Precellys CK01, 0.1 mm). Bead-beating was performed using either the Precellys Homogeniser (Bertin Technologies) at 6,500 rpm for 45 s, the Mini-Beadbeater-24 (BioSpec Products) at 3,000 rpm for three cycles of 1 min with 1-min breaks on ice, or the Qiagen TissueLyser (Qiagen) at 1/30s (1,800 rpm) for 5 min.

Column-based RNA extraction

The PAXgene™ RNA extraction kit (PreAnalytiX, Qiagen) was used following a previously described modified protocol for low-volume (0.5 ml) samples[173]. However, washed bacterial stocks (0.5 ml) were used instead of whole blood. For automated RNA extraction, the Qiagen MagMax Microbiome Kit (Qiagen) and KingFisher Duo automated platform (ThermoFisher Scientific) were used following the manufacturer's instructions.

Guanidine thiocyanate/phenol-based RNA extraction:

TRIzol reagent (ThermoFisher Scientific) and TRI reagent™ solution (Invitrogen™) were used either according to the manufacturer's instructions or using a modified protocol which included a mechanical lysis step by performing bead-beating in TRIzol/TRI reagent™ before phase separation. Lastly, a modified protocol including a heating step at 60°C for 5 min during the initial TRIzol cell lysis incubation was tested (“Modified hot TRIzol protocol”).

RNA clean-up and concentration

The RNA Clean & Concentrator-25 kit (Zymo Research) was utilised following the manufacturer's instructions to purify and concentrate the upper aqueous supernatant after chloroform phase separation in the TRI reagent protocol.

Primers and probes used for host and bacterial gene expression analysis (RT-qPCR)

Supplementary Table 5: Molecular characteristics of in-house designed bacterial and host primers and Multiplex probes. FWD: Forward primer; REV: Reverse primer; bp: base pairs.

	**Target gene**	**Gene description**	**Sequence**		**Length**
Staphylococcus epidermidis	*secA*	Protein trans-locase subunit (ATPase)	FWD	AAATGATTGCTGAGGCGGGA	189 bp
			REV	GATCTCCTTGGCGACCTGAG	
			Probe	GGTGGCTTAGCAGTGATTATTAATGAA	
	gyrB	DNA gyrase subunit B	FWD	GGCCGTGGTATTCCTGTTGA	105 bp
			REV	GTATCCGCCACCTCCGAATT	
			Probe	CTGCTGTCGAAGTTATCTTAACTGTAC	
	psmβ1/2	Phenol-soluble modulin β 1/2	FWD	AGCAGCACAAGATCAAGATTGG	80 bp
			REV	TACCTAATACGCTAACGCCACT	
			Probe	ATTAGGAACTAGTATCGTTGACATCGT	
Staphylococcus aureus	*era*	GTPase	FWD	TCCCTCATGCGATTGGTGTT	160 bp
			REV	CGTCTCGCACGTTTTCCTAC	
			Probe	TATGTTGAAAGAGATTCGCAAAAAGGA	
	gyrB	DNA gyrase subunit B	FWD	TTGGTACAGGAATCGGTGGC	86 bp
			REV	TCCATCCACATCGGCATCAG	
			Probe	CGAAAGCAAGATATCACAAAATCGTCA	
	hlgA	γ-hemolysin subunit A	FWD	TATGGGGCGTCAAAGCGAAT	218 bp
			REV	TTCAAATTCGCTCGTGTCGC	
			Probe	CTTGTACAAAGTGGATTTAACCCTTCA	
Homo sapiens	*HUPO*	Human acidic ribosomal protein	FWD	GGAGACAAAGTGGGAGCCAG	90 bp
			REV	GAACACCTGCTGGATGACCA	
			Probe	CTGCTGAACATGCTCAACATCTC	
	B2M	β-2 Microglobulin	FWD	AGGTGTATGGCCCCAGGTAT	94 bp
			REV	ACCACCTGCCTTTATCCTGC	
			Probe	GCCATATTACTGACCCTCTACAGAG	
	IL1B	Interleukin-β1	FWD	CTTAAAGCCCGCCTGACAGA	167 bp
			REV	ACACTGCTACTTCTTGCCCC	
			Probe	ATTTGGTTCTAAGAAACCCTCTGTCAT	
